# PAV markers in *Sorghum bicolour*: genome pattern, affected genes and pathways, and genetic linkage map construction

**DOI:** 10.1007/s00122-015-2458-4

**Published:** 2015-01-30

**Authors:** Xin Shen, Zhi-Quan Liu, Anne Mocoeur, Yan Xia, Hai-Chun Jing

**Affiliations:** 1 Key Laboratory of Plant Resources, Institute of Botany, Chinese Academy of Sciences, Beijing, 100093 China; 2 Department of Plant and Environment, Faculty of Sciences, University of Copenhagen, 1871 Frederiksberg, Denmark; 3 University of Chinese Academy of Sciences, Beijing, 100049 China

## Abstract

**Key message:**

**5,511 genic small-size PAVs in sorghum were identified and examined, including the pattern and the function enrichment of PAV genes. 325 PAV markers were developed to construct a genetic map.**

**Abstract:**

Presence/absence variants (PAVs) correlate closely to the phenotypic variation, by impacting plant genome sizes and the adaption to the environment. To shed more light on their genome-wide patterns, functions and the possibility of using them as molecular markers, we generated next generation genome sequencing data for four sorghum inbred lines and used associated bioinformatic pipelines to identify small-size PAVs (40–10 kb). Five thousand five hundreds and eleven genic PAVs (40–10 kb) were identified and found to affect 3,238 genes. These PAVs were mainly distributed on the sub-telomeric regions, but the highest proportions occurred in the vicinity of the centromeric regions. One of the prominent features of the PAVs is the high occurrence of long terminal repeats retrotransposons and DNA transposons. PAVs caused various alterations to gene structure, primarily including the coding sequence variants, intron variants, transcript ablation, and initiator codon changes. The genes affected by PAVs were significantly enriched in those involved in stress responses and protein modification. We used 325 PAVs polymorphic between two sorghum inbred lines Ji2731 and E-Tian, together with 49 SSR markers, and constructed a genetic map, which consisted of 10 linkage groups corresponding to the 10 chromosomes of sorghum and spanned 1,430.3 cM in length covering 97 % of the physical genome. The resources reported here should be useful for genetic study and breeding of sorghum and related species.

**Electronic supplementary material:**

The online version of this article (doi:10.1007/s00122-015-2458-4) contains supplementary material, which is available to authorized users.

## Introduction

Single nucleotide polymorphisms (SNPs) and small insertion/deletion polymorphisms (IDPs) have long been thought to be the only means through which most of genetic variations could arise (Feuk et al. 2006). Recently, the central role of structural variation has been gradually emerging through the examination of more sequenced genomes of animals and plants obtained from the next generation sequencing platforms (Tuzun et al. 2005; Kidd et al. 2008; Conrad et al. 2010). Structural variation refers to genomic alterations such as insertions, deletions, duplications, inversions, and translocations covering at least 50 base pairs (bp) (Feuk et al. 2006; Mills et al. 2011). Presence and absence variants (PAVs) are an important type of structural variation present in one genome but entirely missing in the other (Springer et al. 2009), and play an important role in shaping genomes and contribute to phenotypic diversity (Marroni et al. 2014).

PAVs have been widely found in the human genomes (Mills et al. 2011; McKernan et al. 2009; Hastings et al. 2009; Kidd et al. 2008; Korbel et al. 2007; Redon et al. 2006) and have been implicated to cause diseases in humans through the positional effect and the alteration of the gene dosage (Sebat et al. 2007; Zhang et al. 2009). They also contribute to the observed phenotypic variation (Conrad et al. 2010), and determine the fitness with potential evolutionary implications (Stefansson et al. 2005). Compared to the human genome studies, PAVs are less investigated in plants, but are certainly prevalent. For example, in maize (*Zea mays*), it has been reported that 20 % of genome segments (about 10,000 genes or gene fragments) were not shared between inbred lines B73 and Mo17 (Morgante et al. 2005), while an array of comparative genome hybridization (aCGH) showed that more than 1,000 PAVs affected at least 180 single copy genes (Springer et al. 2009). In rice (*Oryza sativa*), it has been reported that 2.2 and 3.3 % of *indica* and *japonica* genes were absent in the corresponding subspecies, respectively (Yu et al. 2005), and 5.2 % of the genes were found with presence and absence polymorphisms between *japonica* Nipponbare and *indica* 93-11 (Ding et al. 2007). A comparison of 18 fully sequenced Arabidopsis (*Arabidopsis thaliana*) genomes showed that on average 775 genes per accession have more than 50 % regions with deletions or polymorphism relative to the reference accession Col-0 (Gan et al. 2011). Furthermore, a comparison of 80 Arabidopsis genomes revealed that 8.9 % of the total genes in *A. thaliana* showed PAVs averaging 444 absent genes per accession (Tan et al. 2012). In other plants, such as soybean (*Glycine max*) and melon (*Cucumis melo*), recent studies also found that a large number of PAVs were distributed in their genomes (Lam et al. 2010; McHale et al. 2012; Gonzalez et al. 2013). Even since the first description of presence and absence polymorphisms in the *RPM1* gene in Arabidopsis (Grant et al. 1998), an increasing number of studies indicated that a dominant feature of the PAV genes is the enrichment in genes involved in the stress responses, especially the disease resistance (Shen et al. 2006; Ding et al. 2007; Ziolkowski et al. 2009; Lam et al. 2010; McHale et al. 2012; Tan et al. 2012; Gonzalez et al. 2013). This feature has also been confirmed to be present in the mammalian (Iafrate et al. 2004; Yalcin et al. 2011), Drosophila (Kern and Begun 2008), and bacteria (Arrach et al. 2008). However, since a large number of fully sequenced individual genomes are the prerequisite for such studies, there is still a lack of a clear estimate of the PAV gene numbers, proportions, and variation patterns.

Sorghum (*Sorghum bicolour*) is the fifth important cereal crop worldwide after wheat, rice, maize, and barley (FAO 2004). It adapts well in the arid and semi-arid areas due to its ability to tolerate drought stress (Dalal et al. 2012). To deflate tradeoff between food and biofuel production, sweet sorghum, a variant of grain sorghum, has been deemed an ideal dedicated biofuel crop due to its high stem sugar and juice accumulation. Sorghum is an interesting system to study changes in genome structure for a number of reasons. It has a relatively small genome (about 730 Mb) and does not seem to have undergone whole-genome duplications as in other closely related crops such as maize, sugarcane, *miscanthus*, and switchgrass (Paterson et al. 2009), making it a more tractable system to establish gene–phenotype associations. Furthermore, as a predominantly selfing plant, sorghum accessions representing elite inbred lines, land races (caudatum, durra, bicolor, guinea, and kafir) of *S. bicolour* ssp. *bicolour* and ssp. *verticilliflorum* (*arundinaceum*, *aethiopicum*, and *verticilliflorum*) have greater linkage disequilibrium (LD) and lower sequence variation than in maize as evaluated with sequences of RFLP markers (Hamblin et al. 2004). This notion is further supported by assays using SNPs and IDPs markers (Bekele et al. 2013; Zheng et al. 2011; Zou et al. 2012; Mace et al. 2013). However, different from the situation in the exotic lines/landraces, the genetic diversity among the publically available inbreds tends to be low (Menz et al. 2004). Sorghum has a very high outcrossing tendency (Barnaud et al. 2008), which could potentially increase the genetic variation. Taken together, these characteristics of sorghum genomes offer a unique niche to study structural variation and its impact on important phenotypic traits.

Molecular markers have been a vital tool in both basic and applied research for fingerprinting genotypes, identifying genetic diversity, defining variety identity, assisting breeding selection and phylogenetic analysis (Vos et al. 1995; McCouch et al. 1997; Kolliker et al. 2001). Since early 1990s, a number of DNA-based molecular markers such as restriction fragment length polymorphism (RFLP ) (Hulbert et al. 1990; Whitkus et al. 1992; Xu et al. 1994; Dufour et al. 1997; Kebede et al. 2001; Bowers et al. 2003; Subudhi and Nguyen 2000), random amplified polymorphism (RAPD) (Dahlberg et al. 2002), amplified fragment length polymorphism (AFLP) (Boivin et al. 1999), simple sequence repeat (SSR) (Menz et al. 2002; Wu and Huang 2007), and diversity arrays technology (DArT) (Bouchet et al. 2012; Mace et al. 2008) have been used for constructing genetic maps and assessing intraspecific diversity in sorghum. Recently, with the advance in sequencing and resequencing sorghum genomes, sequence-based molecular markers such as SNPs have been increasingly exploited for genetic mapping (Bekele et al. 2013; Zou et al. 2012). In contrast, PAV markers attract less attention. PAV markers with moderate sizes have the advantages of simple operation and less demanding for small laboratories (Wu et al. 2013; Wang et al. 2014). In particular, within-gene or genic PAVs are potentially functional markers which are a good “translator” from genomic study into improved crop varieties (Andersen and Lübberstedt 2003).

Previously, we analyzed PAVs with size fragments larger than 30 kb in sorghum and showed that large-size PAVs are widespread in sorghum inbred lines of different origins (Zhang et al. 2014). This prompted us to explore further in detail the small-size (40–10 kb) PAVs. Here we reported our study focusing on the genic small-size PAVs, including the distribution over the genome, the function, and the pathway enrichment of genes affected. We used the available genome resequencing data and identified 5,511 genic small-size PAVs. We showed that these PAVs were preferentially distributed on different parts of chromosome, and the affected 3,238 genes were predominantly involved in stress response and protein modification. As a proof of concept, 325 PAV markers from 10 chromosomes were used to construct a genetic map together with 49 SSR markers, which showed the advantages of genome coverage, integration of physical and genetic maps, and a better functionality over maps constructed with other molecular markers used in sorghum.

## Materials and methods

### Plant materials

Four *S*. *bicolour* inbred lines, including two grain sorghum (Ji2731, BTx623) and two sweet sorghum (E-Tian, Keller), were used for small-size PAVs (40–10 kb) discovery and validation in this study. Among them, the genomes of Ji2731, E-Tian, and Keller were resequenced (Zheng et al. 2011), and the reference genome of BTx623 was previously published (Paterson et al. 2009). These four lines exhibit significant phenotypic variation in plant height, grain yield, photoperiods, stem sugar and juice accumulation, and salt stress tolerance. An F_2_ population consisting of 209 individuals derived from a cross between Ji2731 and E-Tian was developed in Gongzhuling, Jilin province from May to October 2010 and used for genetic linkage group analysis. A 15-row plot of 5 m row length was planted. The inter-row space was 70 cm, and three seeds per hole were sown at 4-cm depth and 20-cm intervals. Full irrigation and timely weeding were carried out as required during the whole growing season, and 225 kg/ha urea was applied followed by irrigation in the week before sowing.

### Sequence data sets

The whole-genome sequences of BTx623 were retrieved from the version of *S*. *bicolour* v1.4 in the Phytozome 9.0 databases (www.phytozome.org) and used as the reference. The sequence data of three sorghum lines (Ji2731, E-Tian, and Keller) previously resequenced to approximately 12× coverage by Illumina 100-bp paired-end sequencing (Zheng et al. 2011) were acquired at *GigaScience* (Zheng et al. 2011; www.gigasciencejournal.com). For the detection of PAVs, short insert-size paired-end reads of three sorghum lines genome were first aligned to the reference genome of BTx623 and their assemblies by SOAPaligner, respectively (Li et al. 2009). The ratios of aligned single-end reads to paired-end reads (S/P ratios) were acquired from the alignment results achieved by SOAPcoverage (http://soap.genomics.org.cn). The overall S/P ratio of identified PAVs was evaluated by calculating the number of mapped paired-end reads with the expected orientation and insert size and the unexpected orientation and insert size. Then by calculating the *P* value using Fisher’s exact test, we tested the significant difference between the S/P ratio of PAVs and the S/P ratio of the whole genome. The PAVs were validated when they meet the conditions of (1) *P* value <0.05 and (2) their depths were consistent with the type of PAV. In this study, the PAVs were supported by at least six paired-end reads. A total of 5,511 small-size PAVs with explicit physical positions and fragment sizes were obtained from the SVs dataset and used for investigation (Table S1.1). The data of transposon elements (TEs) of sorghum were downloaded from Helmholtz-Muenchen plantDB (ftp://ftpmips.helmholtz-muenchen.de/plants/sorghum/) and used for matching PAV sequences.

### DNA preparation and experimental validation of PAVs

**Fig. 1:**
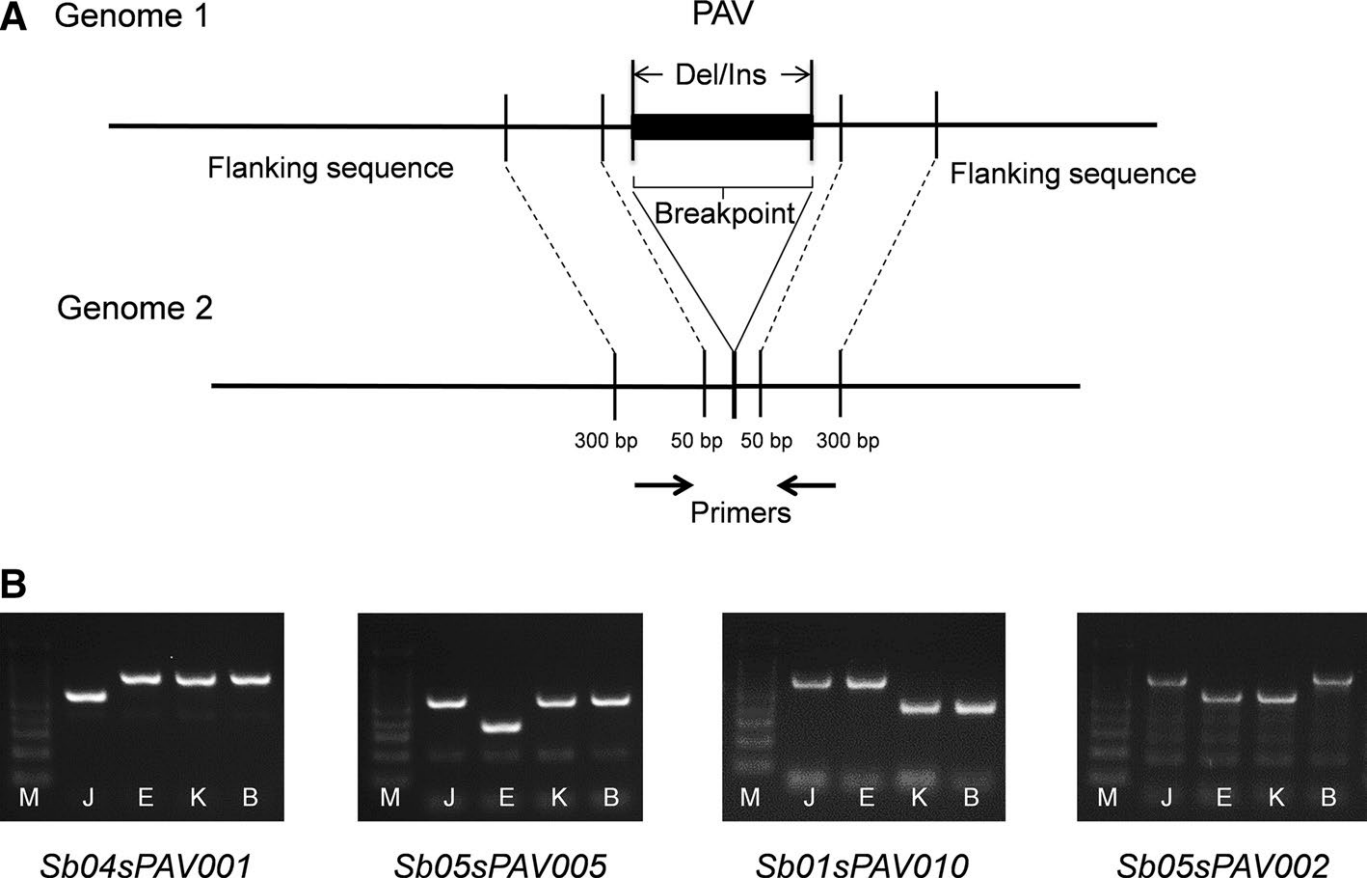
A diagram to show the designing of primers (**a**) and representative gel images (**b**) for the verification of PAVs. Four PAVs between three sorghum lines (Ji2731, E-Tian, and Keller) and BTx623 were presented as examples (*Sb04sPAV001*, *Sb05sPAV005*, *Sb01sPAV010*, *Sb05sPAV002*). A 100-bp DNA ladder marker was on the *left* of the gel and used for size measurement of PCR products

In total, 1,779 PAVs used for experimental validation of polymorphisms between three sorghum lines and BTx623 were randomly selected from the 10 chromosomes of sorghum (Table S1.2). DNA was isolated from young leaves of field-grown plants following a CTAB extraction method (Doyle 1987). Experimental validation of PAVs was executed by polymerase chain reaction (PCR) and agarose gel electrophoresis. The primer pairs used for PCR were designed based on the reference genome sequences of 50–300 bp of up- and down-stream of insertion/deletion breakpoints by the software of Primer 6.0 (http://www.premierbiosoft.com/) (Fig. 1a). A total of 10 μL mixture was used for PCR, containing 1 μL genomic DNA (80–120 ng/μL), 5 μL MasterMix (Biomed, Beijing), 1 μL 10× primer, and 3 μL ultrapure water. The PCR program was set at 94 °C for 5 min, then 34 cycles of 94 °C for 30 s, 55–62 °C for 30 s, and 72 °C for 30 s to 2 min, and followed by a final extension of 10 min at 72 °C. The PCR products were separated on 2–5 % agarose gels depending on the sizes of the fragments and visualized and recorded under UV light.

### Gene ontology enrichment analysis

Two different analyses, including gene ontology (GO) enrichment and Pfam, were carried out on the PAV-affected genes. GO identities (IDs ) of PAV genes and corresponding GO term annotations were obtained from Ensemble Biomart (Smedley et al. 2009). The enrichment of GO terms was assessed using hypergeometric distribution by WEGO (Ye et al. 2006) (http://wego.genomics.org.cn/cgi-bin/wego/index.pl) and agriGO (Du et al. 2010) (http://bioinfo.cau.edu.cn/agriGO/). The detailed information of GO enrichment of PAV genes was presented in Table S1.7. Pfam terms were acquired from Pfam v27.0 (Punta et al. 2012) (http://pfam.sanger.ac.uk/).

### Genetic linkage map construction

In total, 325 PAVs which showed polymorphisms between Ji2731 and E-Tian in validation experiment and 49 SSR markers obtained from the articles published previously (Bhattramakki et al. 2000; Kong et al. 2000; Yu et al. 2010) were selected for developing molecular makers. An F_2_ population of 209 individuals derived from a cross between Ji2731 and E-Tian was genotyped and used for genetic linkage map construction. The software Joinmap^®^ 4.0 (Kyazma, Wageningen, The Netherlands) was applied to construct genetic linkage map by regression mapping algorithm. Because PAV and SSR markers are commonly co-dominant markers, they were pooled together for map construction using the same genotype codes (a/c, b/d, h). In the end, a total of 325 PAV markers and 49 SSR markers (Tables S2.1, S2.2) were assigned to 10 linkage groups using a minimal logarithm of the odds (LOD) threshold value of 3.0 as the criterion, corresponding to the 10 sorghum chromosomes. The Kosambi (1943) mapping function was used to calculate the genetic distance. The graphical representation of the map was drawn by using GGT 2.0 software based on the order and genetic distances of markers (van Berloo 2008). The segregation distortion was estimated using Pearson’s Chi-squared test.

## Results

### Identification of small-size PAVs in sorghum

Resequencing of the genomes of three sorghum lines uncovered a large number of PAVs (Zheng et al. 2011). We previously showed that large-size (>30 kb) PAVs influenced the genome size and varied substantially in sorghum inbred lines (Zhang et al. 2014). We wondered whether the small-size PAVs between 40 and 10 kb within genes are more likely to influence gene function and phenotypic variation. Sequence analysis identified a total of 5,511 genic small-size PAVs affecting 3,238 genes and 9.69 Mb gene sequences (Table S1.1). Among the PAVs identified, 1,635 were commonly shared by the three lines, 777 were shared between Ji2731 and E-Tian, 481 between Ji2713 and Keller, and 620 between E-Tian and Keller (Figure S1). Furthermore, 915, 589, and 494 PAVs were specific to Ji2731, E-Tian, and Keller, respectively.

To test the authenticity of the PAVs obtained from Next Generation Sequencing, we selected 1,779 PAV events with sizes in the range of 100–2,000 bp polymorphic between the reference line BTx623 and the three resequenced sorghum lines for experimental verification (Table S1.2). As shown in Table 1, 313 had no or non-specific amplifications due to primer sequence errors from either the reference genome (BTx623) or the targeted genomes (Ji2731, E-Tian, and Keller). In the end, we had 1,466 clean single PCR products (simple insertion or deletion events), of which 1,142 were consistent between NGS data and PCR results, while 105 were detected in NGS but not in PCRs and 219 were not detected in NGS but detected in PCRs. As such, the overall validation rate is 77.9 %, false positive 7.1 %, and false negative 14.8 %, respectively.

**Table 1:** Summary of experimental validation of NGS PAVs in three sorghum lines

Group	PAV category	Ji2731	E-Tian	Keller	Sub-total
PAVs from sequencing data	PAVs	526	434	17	978
	No PAVs	325	417	60	801
PAVs from PCR results	PAVs				
	Confirmed PAVs	396	305	15	716
	False positives	45	58	2	105
	No PAVs				
	Confirmed no PAVs	156	219	51	426
	False negatives	108	102	9	219
	No or non-specific products	146	167	0	313

**Fig. 2:**
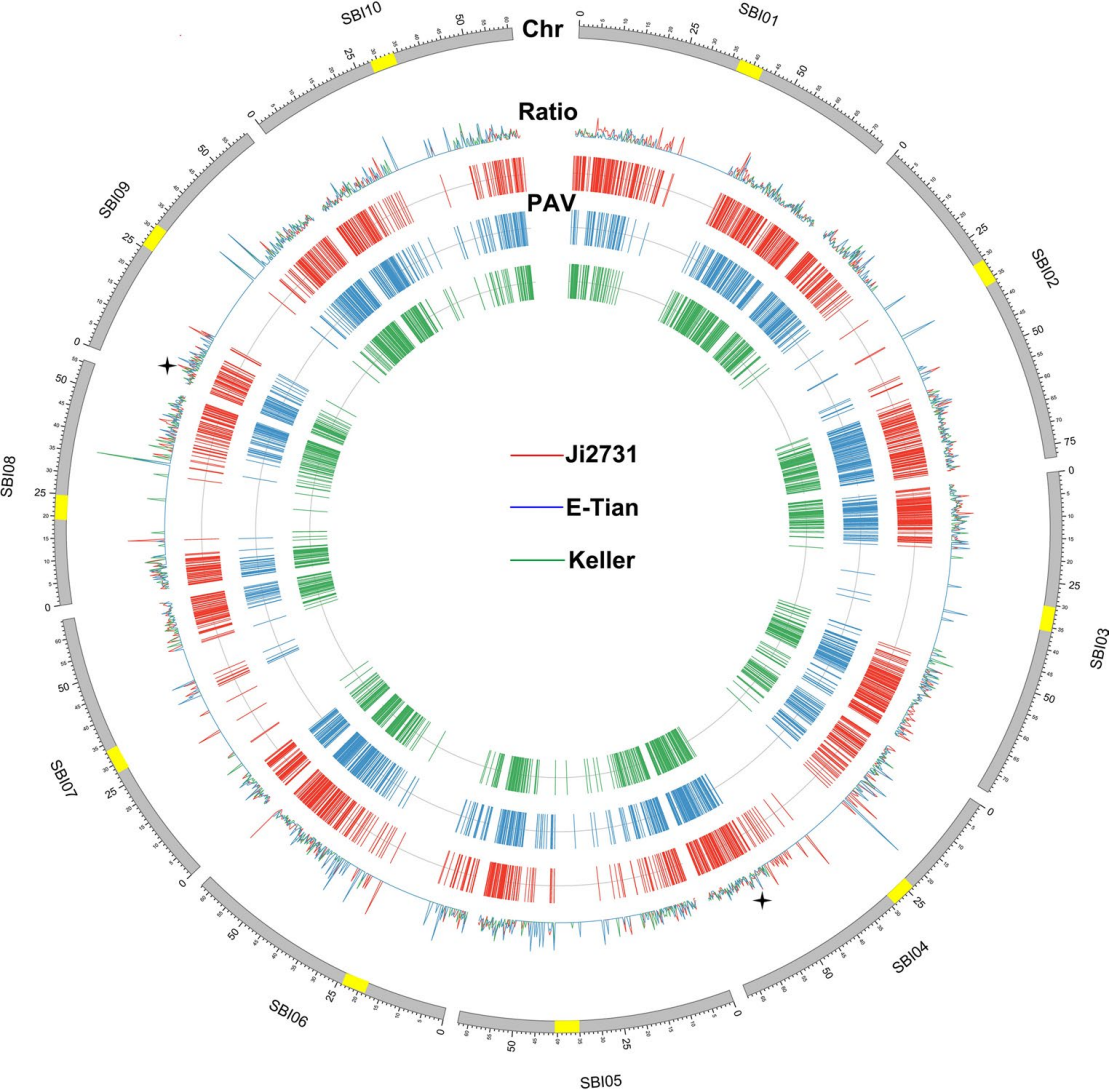
Genome-wide distribution of 5,511 genic small-size PAVs (40–10 kb) discovered from three sorghum lines. The *ratio ring* shows the ratio between the number of genes with PAVs and the number of total genes in the 300-kb bin of the sorghum genome. The *yellow bands* denote the centromeric regions. The *star symbols* stand for the regions with significant enrichment of PAVs by Fisher’s exact test (*P* value <0.05). The ten chromosomes are drawn by a scale of Mb (color figure online)

Figure 2 shows the differential distribution of 5,511 PAVs on the 10 chromosomes. We examined the occurrence of the PAVs in relation to the gene density along each chromosome and found that Chromosome 5 had the highest occurrence with one PAV per 15.67 genes, whereas Chromosome 7 had the lowest with one PAV per 22.18 genes (*P* value <0.05 by Fisher’s exact test) (Fig. 2; Table S1.3). Although the number of PAVs varied within different regions of individual chromosome, the highest proportions of PAVs often occurred around centromeres (Fig. 2; Table S1.4). A 300-kb bin size was used to examine the distribution of PAVs on a finer scale and revealed two incidences of enrichment on Chromosome 4 in Ji2731 (SBI04: 53100001–53400000) and Chromosome 9 in E-Tian (SBI09: 3900001–4200000) (*P* value <0.05 by Fisher’s exact test) containing 11 and 12 PAVs, respectively (Table S1.4).

For the distribution of the fragment sizes of 5511 PAVs, we found that the number of PAVs peaked at the 500–999 bp bin with median value of 670 bp (Figure S2). The smallest group of PAVs fell into the 40–99 bp bin. The insertions were much shorter ranging from 43 to 347 bp when compared to deletions which ranged from 259 to 9,991 bp in length. In most of all next generation sequencing data generated using the Illumina platform, it is routine rather than exception that only short insertions could be detected. Such marked asymmetry might be caused by the use of short reads (<500 bp) and by the detection method/algorithm of PAVs.

### Sequence features and functional categories of PAV genes

To gain insight into the sequence features of small-size PAVs, we analyzed the signature of TEs of PAV sequences. As shown in Table 2, out of the 5,511 PAVs, 3,742 (67.9 %) contained TEs, influencing about 4.5 Mb genomic sequences. In total, 7,532 TEs were found falling into three major categories: retrotransposons (5,280), DNA transposons (2,227) and unclassified (25). Long terminal repeats (LTR) accounted for the largest proportion (98.3 %) of the retrotransposons, while the DNA transposons contained 73.3 % unclassified elements and 26.7 % of the terminal inverted repeats, respectively. Table 2 also shows that limited full-length LTRs were found, while in the DNA transposons, most of them were full-length elements. Furthermore, most of the full-length/complete TEs were found as single events in a PAV, only limited PAVs contained 2, 3, 4, or 5 TEs (Table S1.5). This is consistent with our PCR results showing that most of the PAVs are simple insertion and deletion events.

**Table 2:** Categories of transposon elements in PAVs

Category	Feature	Number	PAVs involved	Genes affected	Sequences (bp)
Retransposons		5280	2823	1745	3502807
LTR	Full length	233	213	128	1149923
	Partial	4957	2634	1644	2317591
LINE	Partial	24	20	13	14303
Unclassified	Partial	66	60	39	20990
DNA transposons		2227	1767	1121	1001317
TIR	Full length	370	354	232	561978
	Partial	225	128	79	98989
Unclassified	Full length	1308	1185	768	276012
	Partial	324	286	201	64338
Unclassified	Partial	25	14	9	5805
Total		7532	3742	2270	4509929

*LTR* long terminal repeats, *LINE* long interspersed nuclear elements, *TIR* terminal inverted repeats

**Fig. 3:**
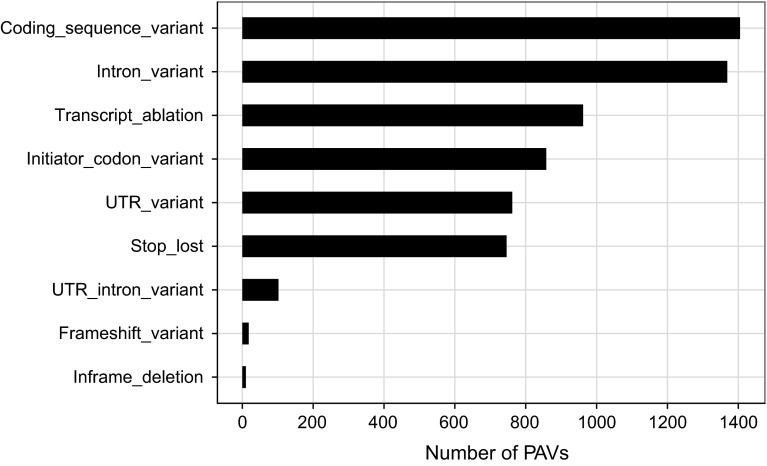
Distributions of PAVs in different variants of gene structure. The *x* axis represents the frequency of PAVs

We analyzed the impact of small-size PAVs on the gene structure. By using Variant Effect Predictor at the Ensemble website (http://plants.ensembl.org/tools.html), nine different alterations in gene structure caused by PAVs were found (Fig. 3). Among them, the coding sequence variants, transcript ablation, initiator codon variants, untranslated region (UTR ) variants, stop-lost and frameshift variants occupied 75.9 % of the total variants, which were proposed to have large effects on the gene function or expression. The remaining variants (intron variants, UTR-intron variants, and inframe deletion) occupied 24.1 %. Therefore, it can be seen that small-size PAVs most likely change gene function or expression by altering gene structure (Table 3).

**Table 3:** Summary of the genetic map constructed with PAV and SSR markers in sorghum

	SBI01	SBI02	SBI03	SBI04	SBI05	SBI06	SBI07	SBI08	SBI09	SBI10	Total	Average
Number of markers	49	42	33	29	34	36	36	37	38	40	374	37.4
PAV markers	41	33	29	26	29	33	32	32	34	36	325	32.5
SSR markers	8	9	4	3	5	3	4	5	4	4	49	4.9
Average genetic distance between markers (cM)	3.87	4.01	4.78	4.58	3.7	3.74	3.65	3.33	3.4	3.21		3.83
Genetic distance (cM)	193.6	168.6	157.7	137.8	125.8	134.7	131.5	123.3	129.1	128.2	1430.3	143.0
Genetic distances (cM) in Mace et al. (2008)	188.1	135.6	83	133.9	130.4	157.1	120.5	184.5	149.3	149.2	1431.6	143.2
Genetic distances (cM) in Bekele et al. (2013)	78	114.1	87.8	113.6	128.1	112.6	94.3	109.9	111.4	135.2	1085	108.5
Physical distance (Mb)	0.86–72.76	0.32–77.31	1.85–67.82	0.42–66.35	0.74–61.17	0.39–62.19	0.13–63.62	0.38–54.74	0.02–58.87	0.12–60.73	640.4	64.0
Physical coverage (%)	97	99	89	97	97	99	99	98	99	99		97

Results from two recent *Sorghum bicolour* genetic maps are compared. LG stands for linkage group

To further understand functional features of PAV genes, we examined GO annotations of the 3,238 PAV genes. In total, 44 unique GO terms (FDR < 0.05) were enriched by WEGO (http://wego.genomics.org.cn/cgi-bin/wego/index.pl) and agriGO (http://bioinfo.cau.edu.cn/agriGO/) (Table S1.7), including 9 cellular components, 16 molecular functions, and 19 biological processes (Figure S3). Furthermore, 105 PAV genes (8.0 %) were involved in cell death, similar to those genes containing large-size PAVs (Zhang et al. 2014). PAV genes related to the reproductive cellular processes and regulation of protein modification, albeit only being 1.8 and 2.8 %, were significantly overrepresented in comparison with the overall frequencies of the corresponding genes (Du et al. 2010), being 0.3 and 0.7 % (*χ* ^2^ = 92.7, *df* = 1; *χ* ^2^ = 74.5, *df* = 1).

**Fig. 4:**
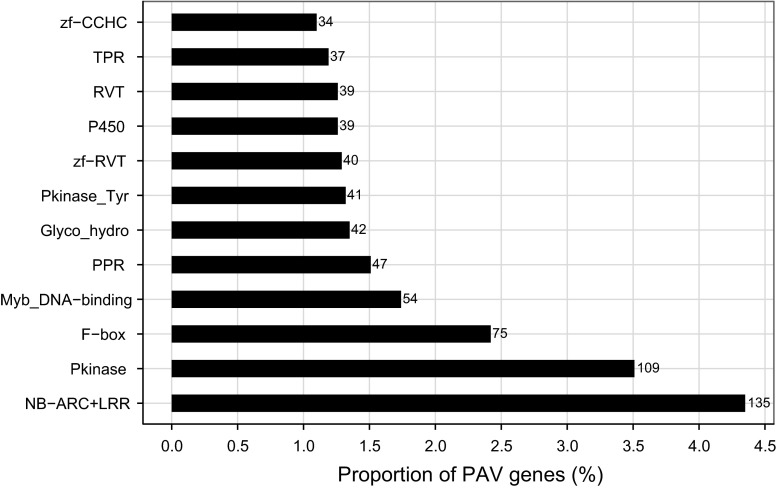
Distributions of PAV genes in the protein families (Pfam). The *x* axis represents the proportion of PAV genes in total PAV genes with annotated Pfam. The *y* axis shows the categories of Pfam. The *numbers after bars* indicate the number of PAV genes

We analyzed protein families and functional domains of PAV genes by Pfam database v27.0 (Punta et al. 2012). In total, 3,105 (95.9 %) PAV genes were classified into 2,529 Pfam categories (Table S1.8). Although most of PAV genes tended to be fragmented across these categories, as shown in Fig. 4, the disease resistance genes with NB-ARC domains and LRR domains were significantly (*χ* ^2^ = 73.5, *df* = 1) overrepresented, accounting for 4.3 % of total PAV genes. Moreover, these resistance genes were predicted to be predominantly involved in the biological process of programmed cell death, receptor signaling, and phosphorylation (FDR < 0.05) (Table S1.9).

### Construction of genetic map by PAV markers

To develop PAVs into functional markers for genetics and breeding of sorghum, we selected 360 PAVs with identifiable polymorphism between Ji2731 and E-Tian from the experimental validation results. To avoid the overlap in the genetic position, in the end, 325 PAV markers were used for the construction of a genetic map (Table S2.1). An F2 mapping population with 209 individuals was obtained by crossing Ji2731 and E-Tian and screened for the construction of genetic map. For constructing linkers between PAVs markers and other molecular marker systems, 192 SSR markers originated from previous genetic maps of sorghum (Bhattramakki et al. 2000; Kong et al. 2000; Yu et al. 2010) were examined and 49 (25.5 %) of them were polymorphic between Ji2731 and E-Tian (Table S2.2).

**Fig. 5:**
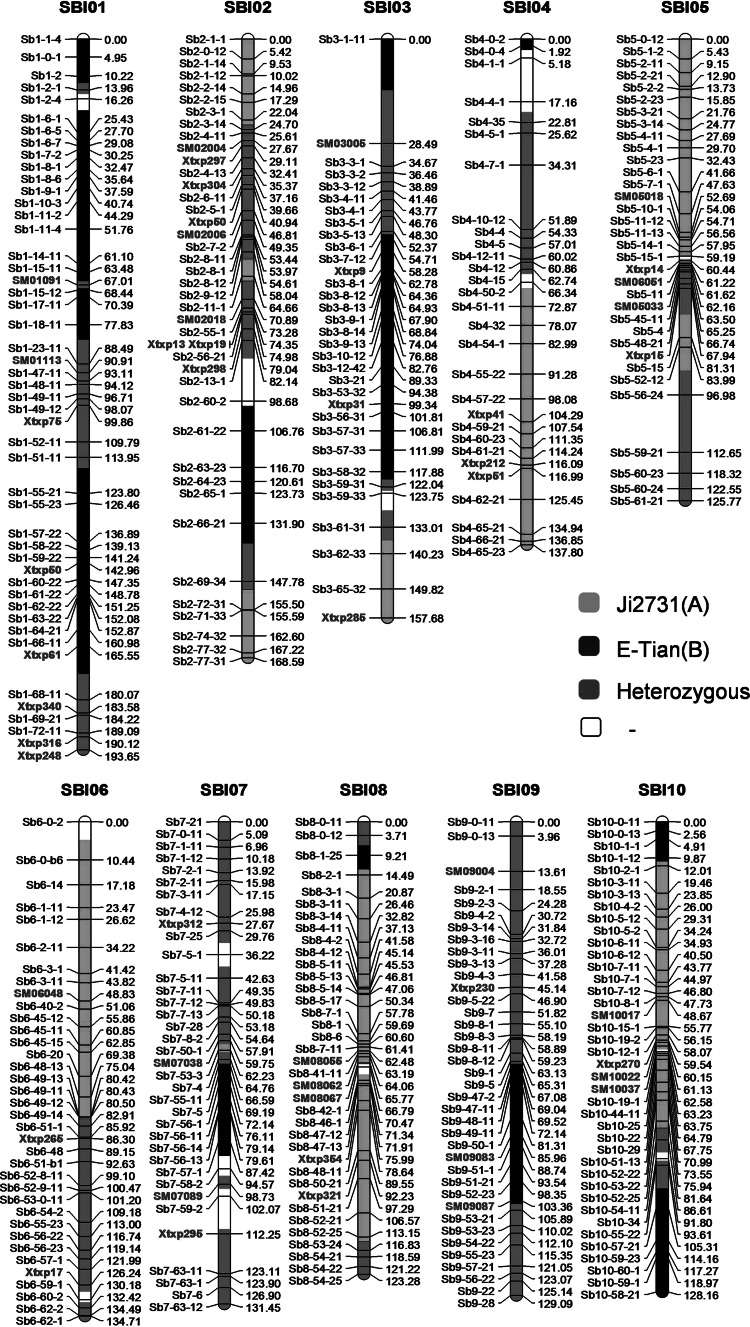
An integrated genetic linkage map of *Sorghum bicolour*. The *vertical bars* represent the chromosomes of *Sorghum bicolour*. The *codes on the left* are the PAV marker loci and the SSR marker loci which are labeled with *red color*, while the corresponding accumulative genetic distances (cM) are on the *right*. The discrete segments of the vertical chromosomal bars are *color*-*coded* according to the allele colors in the figure legends. Heterozygous stands for alleles heterozygous for parents (Ji2731 and E-Tian) alleles. Ji2731(A) is female allele, while E-Tian(B) is male allele. – is missing value (color figure online)

**Fig. 6:**
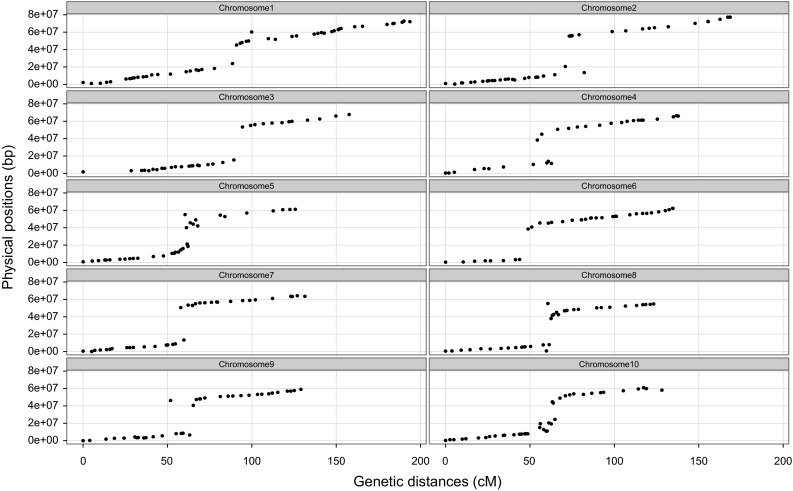
Colinearity between the physical position and the genetic distance of PAV and SSR markers. The *x* axis shows the accumulative genetic distance (cM) in the genetic linkage map. The *y* axis represents the physical positions (bp) obtained by aligning with the reference genome of BTx623

As shown in Fig. 5, a total of 374 markers including 325 PAV markers and 49 SSR markers were assigned to 10 linkage groups, corresponding to the 10 chromosomes of sorghum. This genetic map spanned 1,430.2 cM, with an average marker distance of 3.83 cM and an average PAV marker distance of 4.40 cM. SSR markers interspersed among the PAV markers in each linkage group. According to the physical locations of PAV markers, this map covered 640.44 Mb, which is approximately 97 % of the sorghum genome released from Phytozome v9.1 (http://www.phytozome.org/) with an average physical distance of 1.76 Mb per marker. Nonetheless, on this genetic map, a total of five gaps over 15 cM were encountered on Chromosomes 2 (82.14–98.68 cM; 131.90–147.78 cM), 3 (0.00–28.49 cM), 4 (34.31–51.89 cM), and 5 (96.98–112.65 cM), respectively. We checked the colinearity between the physical and genetic orders of markers. Although the linkage analysis was executed by regression mapping, which was recognized to reduce the conservation of the physical and genetic marker orders compared to the maximum likelihood mapping (MLM) (Cheema and Dicks 2009), the physical and genetic orders of markers on this genetic map were mostly consistent, with little discrepancy by several PAV markers, including one each on Chromosomes 1, 2, and 9, three each on Chromosomes 4 and 8 and four each on Chromosomes 5 and 10 (Fig. 6).

We used Chi-squared test to examine the segregation pattern of all the 374 markers. A total of 112 (104 PAVs and 8 SSR) markers were significantly distorted from the expected Mendelian segregation ratio (*P* value <0.05) (Figure S4; Table S2.3). A detailed exam showed that the majority of the markers with distortion resided on Chromosomes 1 (65.3 %), 2 (38.1 %), 7 (41.7 %), 8 (40.5 %), and 9 (31.6 %), respectively, and tend to form clusters (Figure S4).

## Discussion

Intraspecies structural variations in genes have been proposed to play an important role in the adaptation of particular populations to variation in environmental conditions (Feuk et al. 2006). Here we identified 5,511 small-size PAVs affecting 3,238 genes representing 9.5 % of the total genes in sorghum. This proportion is similar to that of the genes with presence and absence polymorphism in Arabidopsis (Tan et al. 2012). It seems that small-size PAVs influence gene sequences greatly in plants.

### Small-size PAVs distribution and sequence features

The distribution of PAVs on the chromosomes has been investigated in Arabidopsis and been shown that the number of large InDels (>100 bp) along chromosomes increased gradually from telomere toward centromere (Ziolkowski et al. 2009). Conversely, PAVs were mainly accumulated at the distal ends of chromosomes and few on the pericentromeres (Tan et al. 2012). In sorghum, we found that genic PAVs more often resided in sub-telomeric regions and few were around the centromeres (Fig. 2), which may be related to differences in the gene lengths and recombination frequencies in these two regions. In sorghum, high frequencies of recombination were observed on both the distal ends of chromosomes (Mace and Jordan 2011), and high levels of linkage disequilibrium (LD) have been showed in the heterochromatic regions surrounding the centromeres with a recombination suppression rate of 33 % (Kim et al. 2005). Interestingly, the highest ratios of PAV genes to the total genes were found near the centromeres (Fig. 2), which have also been described in Arabidopsis (Tan et al. 2012). Despite centromere has the conserved function as the site for kinetochore formation and sister chromatid join, the DNA sequences associated with the centromeres are highly variable containing many satellite repeats and transposons (Lee et al. 2005; Murphy et al. 2005), which might cause widespread gene conversion (Shi et al. 2010) and variation in the tandem repetitive sequences in the centromere (Miller et al. 1998; Zwick et al. 2000). It is anticipated that such dynamic changes in the DNA sequences may generate high frequency of insertion or deletion.

One of the prominent sequence features of the small-size PAVs is the enrichment of transposable elements (TEs) and repeat sequences. Since its first discovery in maize (Mcclintock 1948), transposable elements have been shown to be prevalent in plant genomes, particularly revealed through the recent whole-genome sequencing of a number of species (Tenaillon et al. 2010). Since TEs can cause duplication, deletion, transposition of nearby non-TE genes or ectopic recombination by a variety of mechanisms, it has been recognized that transposons have a great impact on the genome structure and gene function in nearly all organisms (Kidwell and Lisch 1997; Bennetzen 2000; Wang et al. 2013). In this study, 67.9 % of PAVs contained TEs, affecting 4.5 Mb sequences out of the total 9.69 Mb PAV sequences (Table 2; Table S1.5). Moreover, the proportions of retroelement and DNA transposon varied. The overall ratio of PAV sequences between them was 3.5, which is much lower than that in the whole genome of sorghum (7.3) (Paterson et al. 2009). Within the full-length TEs, 1,678 DNA transposons resided in 1,504 PAVs, whereas only 233 retrotransposons resided in 213 PAVs (Table 2). It seems that DNA transposons might participate in the generation of the small-size PAVs actively. And these features were also found in the large-size PAVs of sorghum in our previous study (Zhang et al. 2014). It is known that DNA transposons transfer sequences by a cut and paste mechanism, whereas LTRs by a copy and paste mechanism. In this study, we found a lot more deletions than insertions, this could be partially due to experimental procedures and the algorithms and the bioinformatic pipeline used, but could also be related to the high occurrence of DNA transposons in the PAVs examined. Overall, our results imply that TEs might play a key role in the formation of PAVs in sorghum.

### Functional features of PAV genes

More attention was given to the functions of genes with structural variation due to their tight relations with phenotypic variation and development of plants. Previous research on PAVs of plants showed that the most predominant feature of the function of the PAV genes was stress response, particularly disease resistance (Ziolkowski et al. 2009; Tan et al. 2012; Gonzalez et al. 2013; Swanson-Wagner et al. 2010; McHale et al. 2012; Bush et al. 2014). Similarly, we showed that the genes for nucleotide binding and protein modification by ubiquitination were enriched (Figure S2), and the NB-LRR genes occupied the largest portion (Fig. 4). This feature was also found within the SNP, small InDel, and large-size PAVs in sorghum (Zheng et al. 2011; Zhang et al. 2014). Such significant enrichments in gene functions and annotated domains have been proposed to reflect the adaptive role of large polymorphic deletions (Bush et al. 2014). However, the proportion of NB-LRR genes with PAVs was not excessively large compared to previous findings in plants (McHale et al. 2012; Tan et al. 2012).

### The feature of the PAV genetic map and segregation distortion

A genetic map, composed of PAV markers and SSR markers, was constructed using an F_2_ population, which was derived from a cross of two resequenced sorghum lines (Ji2731 and E-Tian). This map spanned 1,430.3 cM with average markers distance of 3.83 cM and had average 97 % physical coverage of genome. Compared to the recently published sorghum genetic maps using SNPs and DArT markers (Bekele et al. 2013; Mace et al. 2008), our current map has a larger or approximate genetic distance and physical coverage (Table 2). Moreover, this map with the PAVs markers derived from genes with function annotation might benefit future QTL analysis to quickly establish gene–trait association. The physical positions of markers corresponded to their genetic map orders. Nevertheless, a few disordered markers were distributed on the chromosomes except chromosomes 3, 6, and 7. This phenomenon was also presented in genetic map of sorghum and other plants (Bekele et al. 2013; Ganal et al. 2011; Felcher et al. 2012; Sim et al. 2012; Zhang et al. 2012). It is likely to be caused by the different algorithms for construction of genetic map or the partially inversion of chromosome regions (Felcher et al. 2012; Bekele et al. 2013). Because the recombination rates are suppressed around centromeric regions, it was made that although there is no markers on these regions, the genetic orders were continuous (Fig. 6).

Strongly distorted segregation was detected during the constructing of our current genetic map. 29.9 % of all the markers across the 10 chromosomes showed allele frequencies skewed from their Mendelian expectations (Table S2.3). We found that relatively high proportions occurred prominently on Chromosomes 1, 2, 7, 8, and 9 with the range of 31.6–65.3 %. Some skewed markers were clustered on the long arms of Chromosomes 1 (0–93 cM) and 7 (49–87 cM), respectively (Figure S3). This phenomenon has been reported previously in sorghum and was referred to as the segregation distortion region (SDR) (Murray et al. 2008; Menz et al. 2002; Mace et al. 2009). A recent report confirmed Chromosomes 1 and 7 to be gathered with skewed markers (Felderhoff et al. 2012). Yet, another study further showed that Chromosomes SBI-04 and SBI-08 had more than 50 % of chromosomal regions with segregation distortion (Mace et al. 2009). Previous proposals indicated that these distortions were likely caused by the ablation of gametes or zygotes by a lethal factor (Qi et al. 2004; van Os et al. 2006; Menz et al. 2002) and further study is required to explore the molecular genetics mechanisms.

#### **Author contribution statement**

Hai-Chun Jing conceived and designed the experiments. Xin Shen, Zhiquan Liu, and Anne Mocoeur constructed the genetic map. Xin Shen and Yan Xia analyzed the data. Xin Shen and Hai-Chun Jing wrote the first and the final draft.

## Electronic supplementary material

Below is the link to the electronic supplementary material.

Supplementary material 1 (DOCX 17 kb)

Supplementary material 2 (PDF 383 kb)

Supplementary material 3 (PDF 6 kb)

Supplementary material 4 (PDF 527 kb)

Supplementary material 5 (PDF 9 kb)

Supplementary material 6 (XLSX 2773 kb)

Supplementary material 7 (XLSX 83 kb)
