# Supplementary material for: PAV markers in Sorghum bicolour: genome pattern, affected genes and pathways, and genetic linkage map construction
Source: Theor Appl Genet. 2015 Jan 30;128(4):623–37. doi: 10.1007/s00122-015-2458-4 (PMC4361761; doi:10.1007/s00122-015-2458-4)
Supplement: Supplementary file 1 — Supplementary material 1 (DOCX 17 kb) [file 122_2015_2458_MOESM1_ESM.docx]

**Figure**

**Figure S1.** A Venn diagram showing the shared and unique PAVs amongst three sorghum lines idenfied through next-generation sequencing analysis.

**Figure S2.** Distribution of the fragment sizes of PAVs. The x axis represents five intervals of PAV lengths, 40-99bp, 100-499bp, 500-999bp, 1000-4999bp and 5000-9999bp, respectively. The y axis is the frequency of PAVs in five intervals. The grey and black colors stand for deletion and insertion, respectively.

**Figure S3**. Function enrichment of PAV genes. The x axis shows the functions belonging to three categories of gene ontology (GO). The y axis denotes the percentage of genes with PAVs. a: Function enrichment in the cellular component; b: Function enrichment in the molecular function; c: Function enrichment in the biological process.

**Figure S4.** Distribution of χ^2^ values for each PAV and SSR marker along 10 chromosomes of *Sorghum bicolor*. The black points stand for the markers with the distort segregation diverged from the Mendelian segregation, whereas the grey points represent the markers with the expected Mendelian segregation. The x axis shows the physical positions of markers. The y axis denotes the χ^2^ value of markers.

**Table**

Table S1

Table S1.1. The information of 5511 small size PAVs (40 bp-10 kb) in three sorghum accessions. These PAVs were estimated by resequencing and located in the different regions of genes.

Table S1.2. The detail information of 851 PAVs for the experimental verification of polymorphism between three sorghum lines and BTx623.

Table S1.3. Distribution of 5511 small-size PAVs in ten chromosomes of three sorghum lines.

Table S1.4. Distribution of PAVs in each 300-kb bin of genome. The ratio is obtained by the comparison between PAV gene count and total gene count in the same bin.

Table S1.5. The transposon elements (TEs) analysis of PAV sequence.

Table S1.6. The information of variant type of gene structure caused by PAVs.

Table S1.7. The detail information of GO annotations for 2250 genes affected by PAVs.

Table S1.8. The detail information of Pfam annotations of PAV genes

Table S1.9. The biological processes of 103 resistance genes affected by PAVs.

Table S2

Table S2.1. The detail information of 325 PAV markers involving in the genetic linkage map.

Table S2.2. The information of 49 SSR markers involving in the genetic linkage map.

Table S2.3. Genetic and physical positions of 374 PAV and SSR markers in the genetic linkage map. Segregation of three genotypes was tested by Pearson's Chi-squared Test.
