# Supplementary figures and images for: PAV markers in Sorghum bicolour: genome pattern, affected genes and pathways, and genetic linkage map construction
Source: Theor Appl Genet. 2015 Jan 30;128(4):623–37. doi: 10.1007/s00122-015-2458-4 (PMC4361761; doi:10.1007/s00122-015-2458-4)

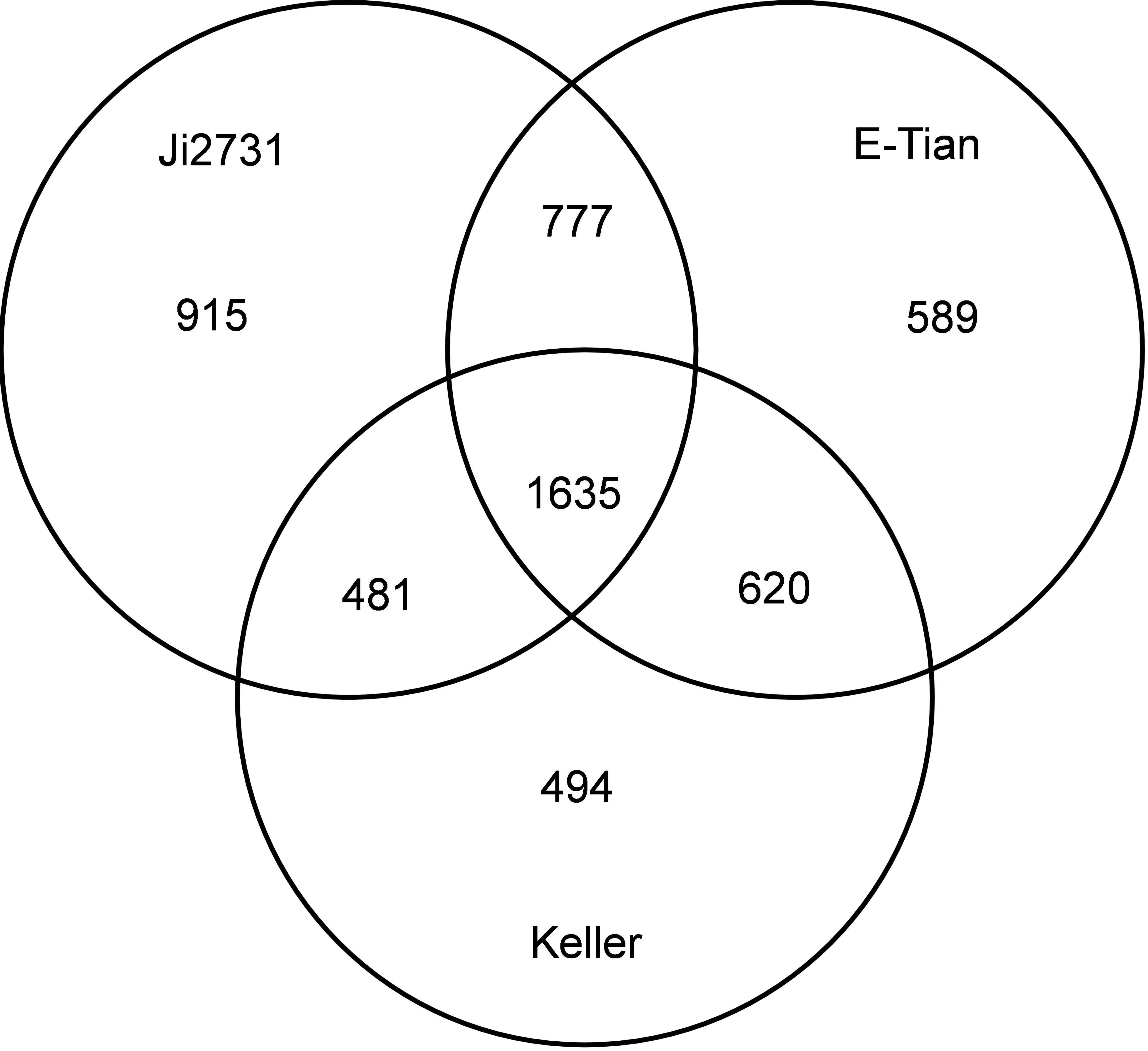

Supplement: Supplementary file 2 — Supplementary material 2 (PDF 383 kb) [file 122_2015_2458_MOESM2_ESM.pdf]

Number of PAVs

300

200

100

0

0

500

1000

PAV Lengths

5000

10000

Deletion  
Insertion

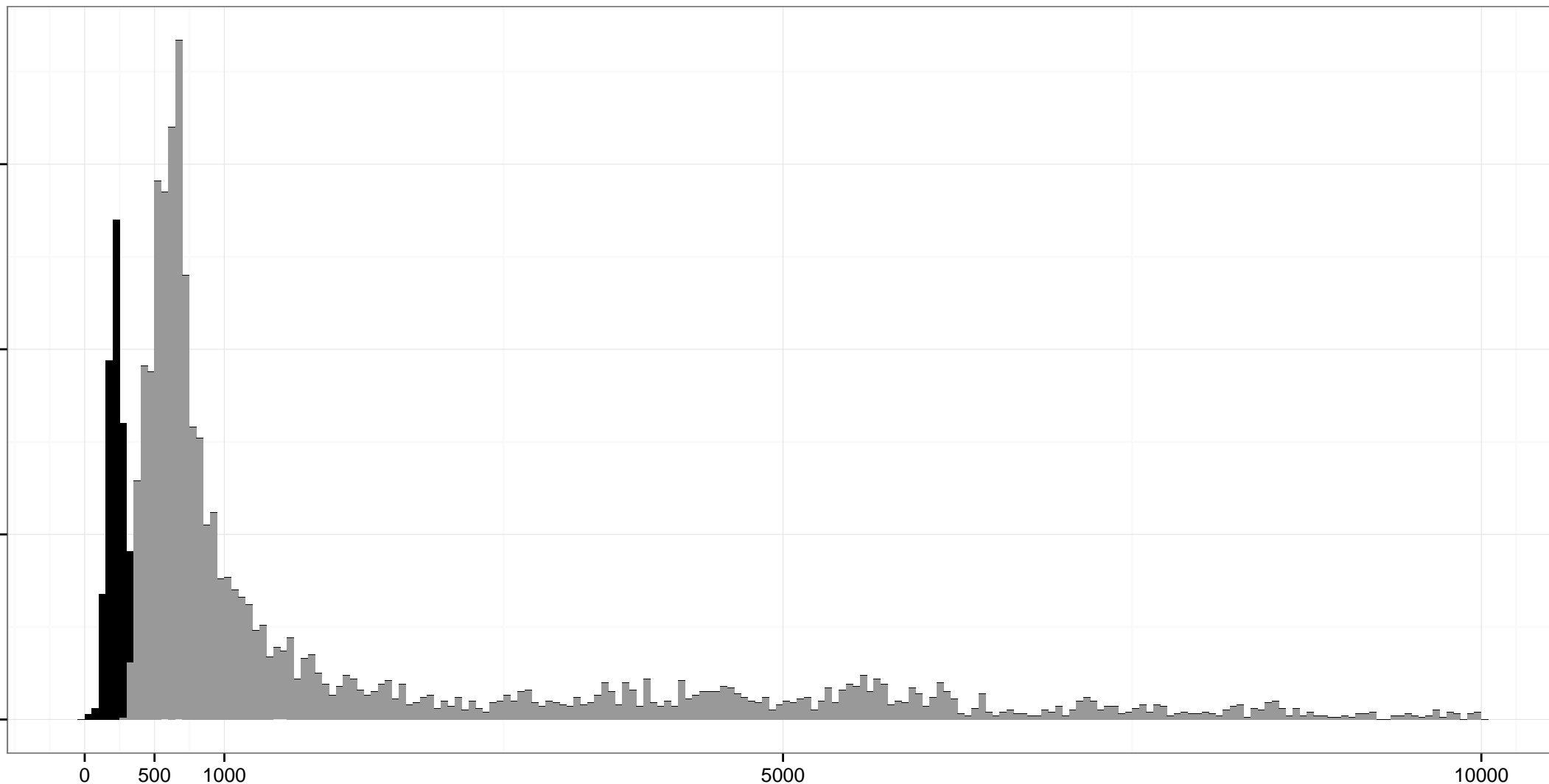

Supplement: Supplementary file 3 — Supplementary material 3 (PDF 6 kb) [file 122_2015_2458_MOESM3_ESM.pdf]

**a**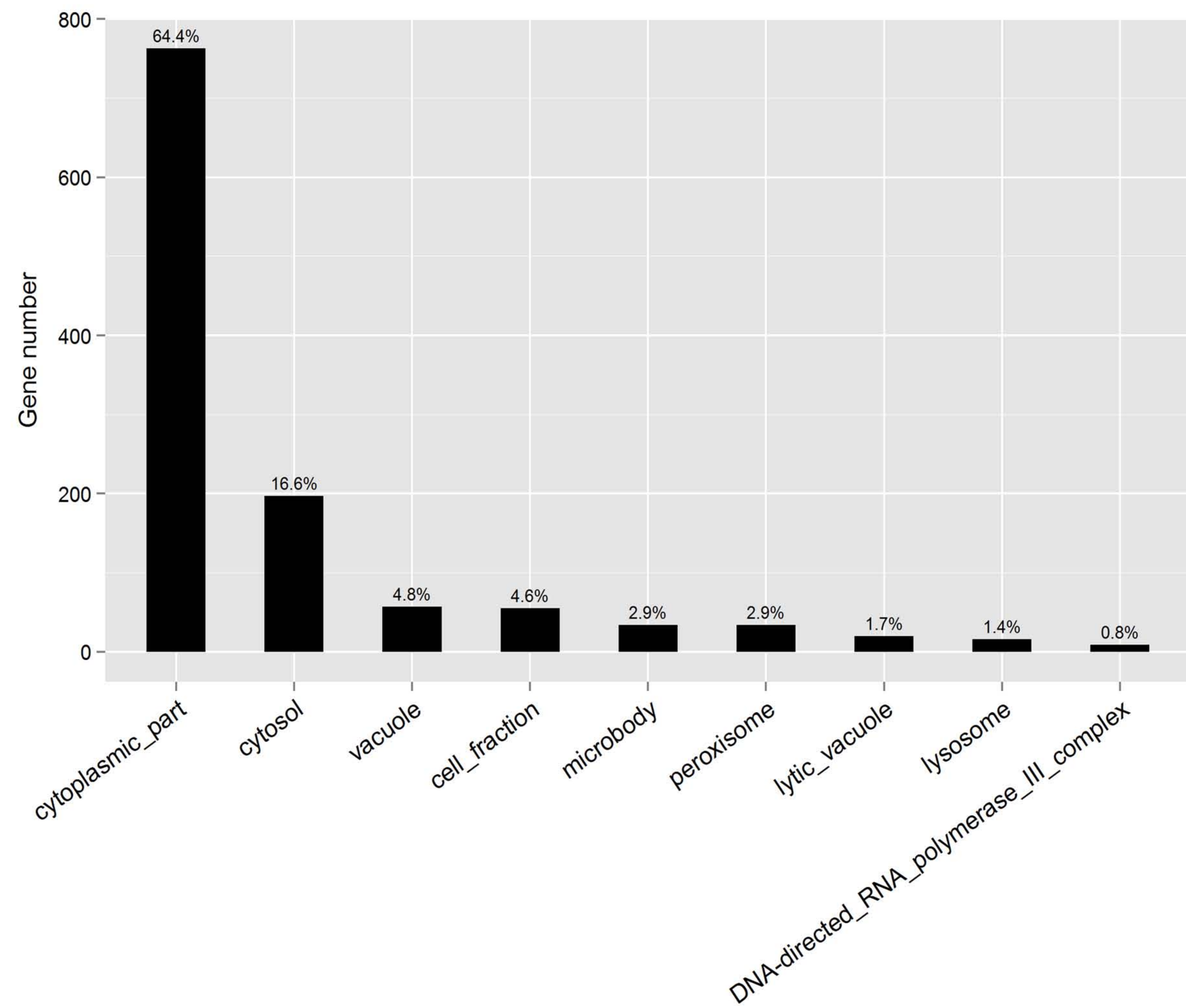**b**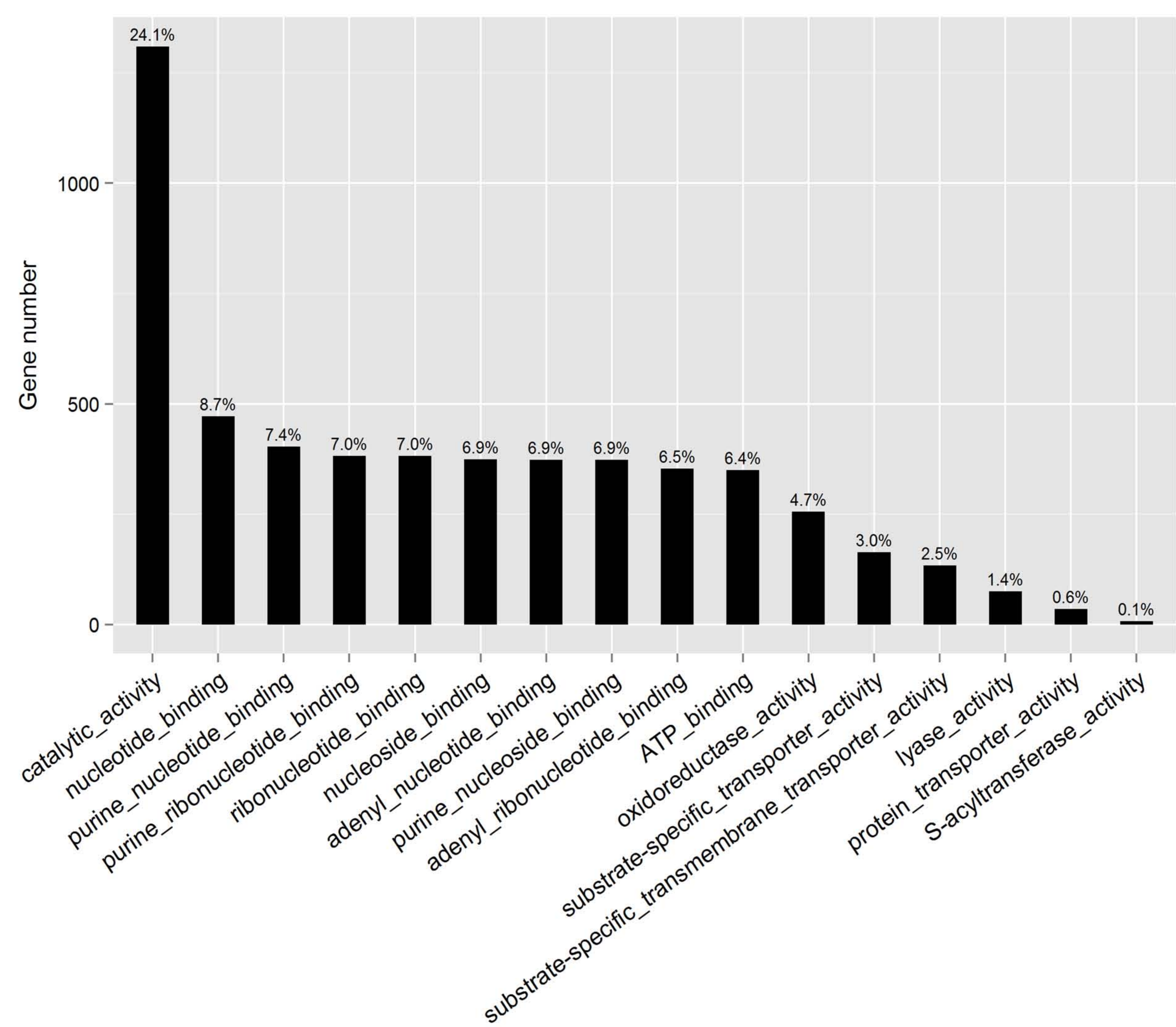**c**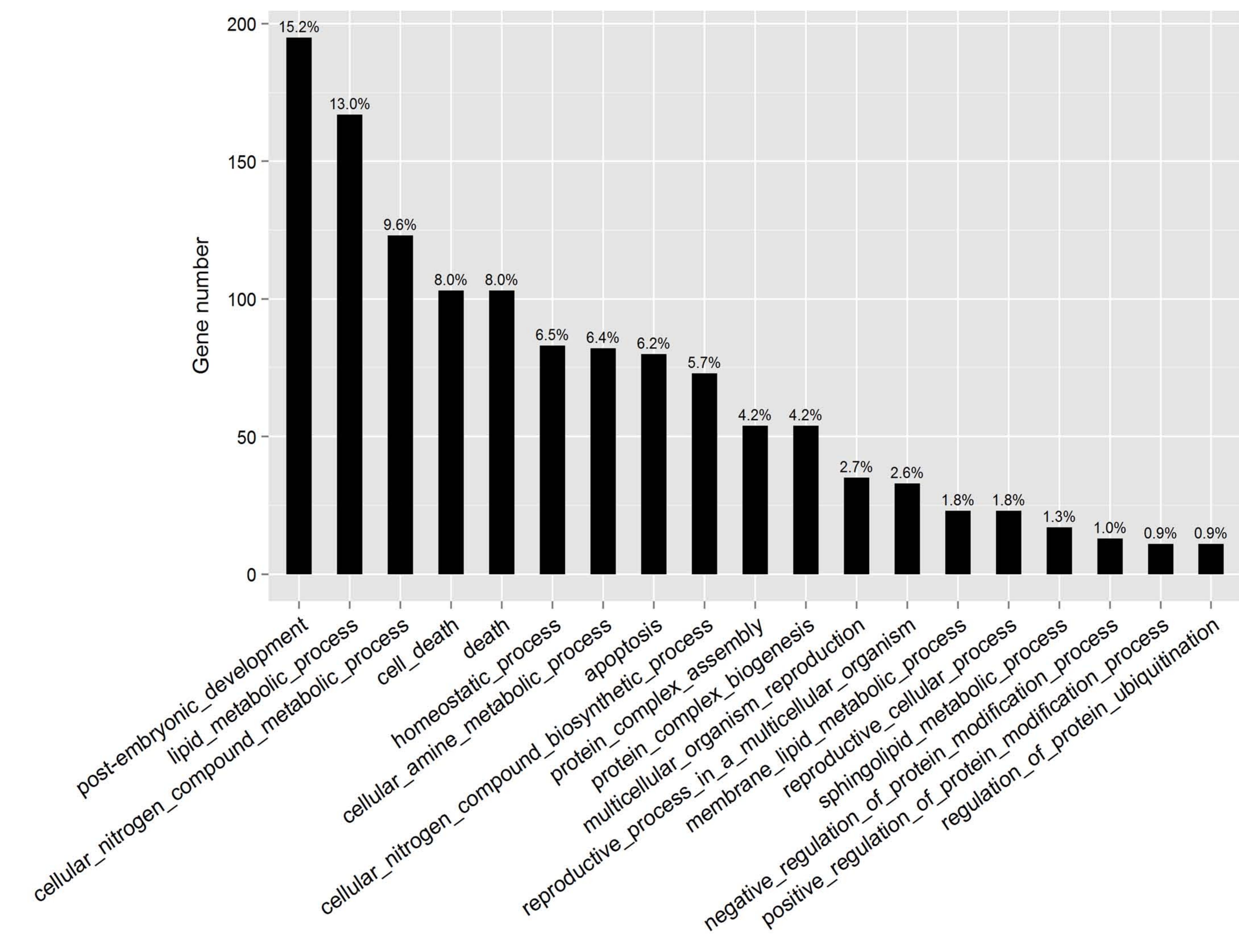

Supplement: Supplementary file 4 — Supplementary material 4 (PDF 527 kb) [file 122_2015_2458_MOESM4_ESM.pdf]

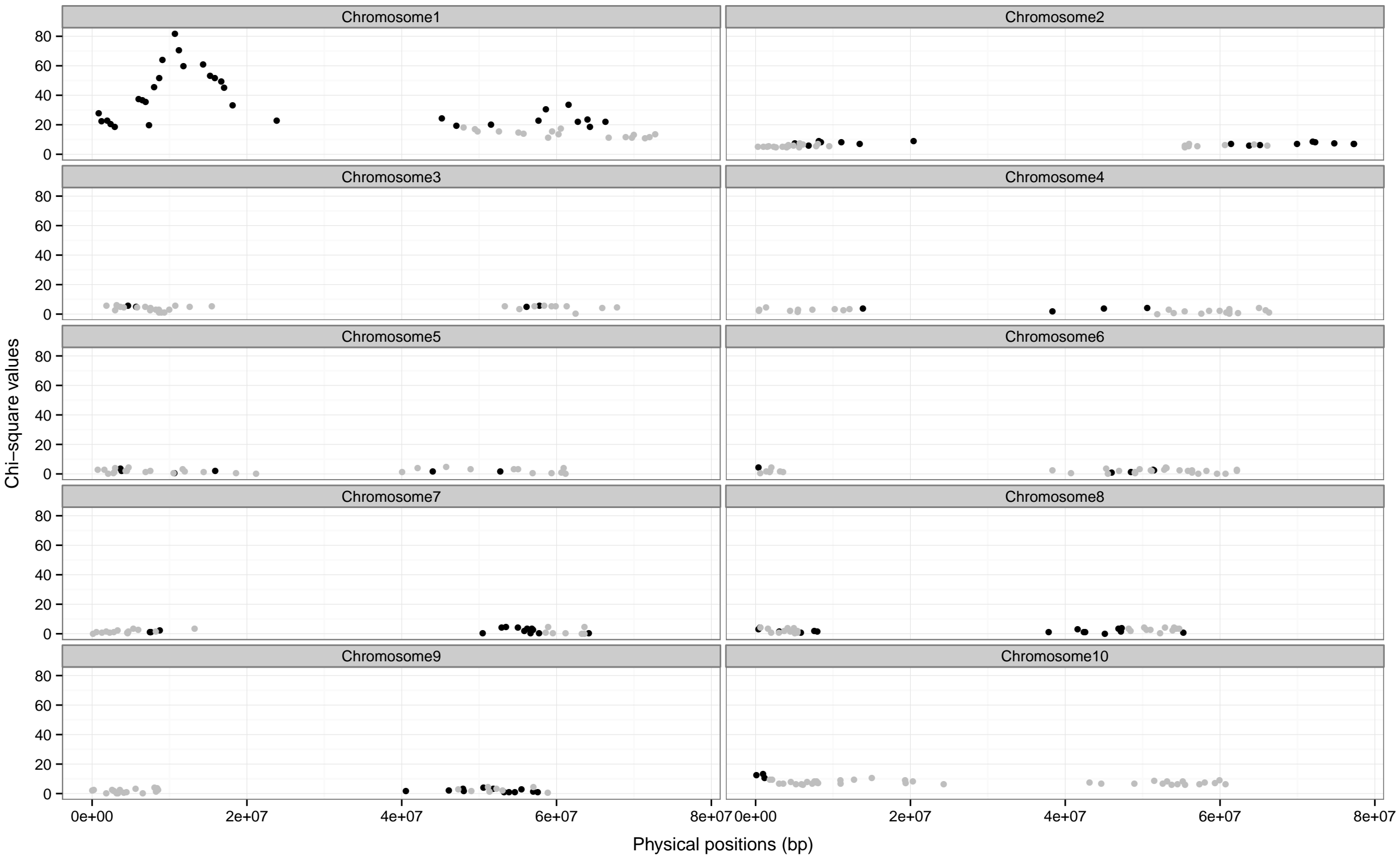

Supplement: Supplementary file 5 — Supplementary material 5 (PDF 9 kb) [file 122_2015_2458_MOESM5_ESM.pdf]
